# Food plants used during traditional wrestling in Kabyè land of Togo

**DOI:** 10.11604/pamj.2016.23.25.7719

**Published:** 2016-02-03

**Authors:** Tchazou Kpatcha, Amegnona Agbonon, Messanvi Gbeassor

**Affiliations:** 1 Laboratoire de Physiologie/Pharmacologie, Faculté des Sciences, Centre de Recherche et de Formation sur les Plantes Médicinales (CERFOPLAM), Université de Lomé BP 1515 Lomé, Togo

**Keywords:** food plants, traditional wrestling, Kabyè land

## Abstract

**Introduction:**

In the traditional sports like the fight, natural products from minerals, animals and plants are used to increase physical resistance and performance. For a better understanding of this practice, an ethnopharmacological survey was carried out in kabyè land, North of Togo, to identify current plants used as foods plants during traditional wrestling.

**Methods:**

Ethnopharmacological data were collected through semi-structured method and personal interviewsin the Kabyè locality during traditional wrestling. At least, twelve villages were surveyed in the study.

**Results:**

Results indicated that 57 plants are widely used by local people as food plants generally during wrestling time. These plants are used traditionally for many others purposes.

**Conclusion:**

We concluded that these plants may serve as sources for pharmacological investigations in physical performance improvement.

## Introduction

Medicinal plants have been an integral part of life in many communities both for food and drug [1]. In many African communities, collection and consumption of wild plant from agricultural and non-agricultural ecosystems has been documented in multiple cultural contexts, illustrating their use and importance among farming households throughout the world [2]. The evidence to date suggests that gathering by farmers occurs in various environments, ranging from intensively farmed areas, to more subsistence oriented horticultural systems, and finally in more pristine areas such as forests. Throughout worldwide and particularly in poor countries, people depend on the forest resources for several purposes like for forest products, medications, food, etc [3]. Many plants have been studied with the aims to improve the Physical performance, but only some of them have been defined to use as stimulant to recover after intense efforts and with anti-fatigue effects [4]. The plants which have shown positive results in scientific studies, with the purpose of improving physical performance, were: Rhodiola rosea [5–10], Eleutherococcus senticosus [11], Schisandra chinensis [12], Panax ginseng [13, 14]. Other plants with similar effects are Rubus coreanus [15], Pseudosasa japónica [16, 17], Chines bamboo [18], Anoectochilus formosanus [19], Camellia sinensis [20] and Allium sativum [4, 21]. Extracts from these plants and several adaptogens complexes, as Erkang [22], also showed improvements in mental resistance, greater concentration and physical development [12, 23, 24]. The Kabyè people of Togo, mostly dependent on nature for their livelihood and they use plenty of wild plants as vegetables in their daily food. Despite the vast knowledge of medicinal plants from Togo, a few attempts have been carried out to document ethnobotanical knowledge in this locality. Some researchers have investigating the traditional pharmacopoeia and medicinal plants in different areas of the country [25–31]. Although these investigations, no studies are undertook on food plant used in perspective to increase physical performance in sportsmen. The aim of this study was to explore and identify some important plant species currently use as food or medicinal plants during traditional wrestling by young sportsmen.

## Methods

### Ethnobotanical data

The study was undertaken during three months (June-August) 2011 by conducting survey in twelve villages throughout the area of Kara. A total of 154 informants were interviewed, of which 96 were wrestlers (age 18-25 years), 10 herbalists and traditional healers (age 27-83 years) in 12 localities. Repeated queries were made to get the data conformed. The preparation of some foodsitem were observed, tasted and documented. Ethnobotanical data were collected using questionnaire as suggested by Jain and Goel [32]. Moreover, interviews and discussions in their localdialect to seek the following information about plants: local name, plants part(s) used. Regarding to taxonomy and phytogeographical types, plant species were systematically identified by the Department of Botany (Faculty of Sciences, University of Lome - Togo). The plant specimens were preserved at the herbarium of the faculty using technique previously described [33].

## Results

### Ethnobotanical findings

The present investigation in Kabyès communities, recorded 57 plants species (Table 1, Table 2, Table 3, Table 4, Table 5) which are used currently during festivals of wrestling. Those species are enumerated below along with their botanical names, plant families, local names, part used and traditional indications (anti-fatigue or antistress uses). The results obtained showed that foods plants are still widely used by the population of Kabyè land during traditional festival of wrestling. The data gathered with the informants during the discussions or during the field trips indicate the high level of knowledge of the traditional healers in this region. The botanical investigation showed that the three most represented families are Anacardiaceae (3 species), Bombaceae (3 species), Zingiberaceae (3 spieces),followed by fourteen families with two species (Alliaceace, Asteraceae, Caesalpiniaceae, Euphorbiaceae, Fabaceae, Lamiaceae, Malvaceae, Poaceae, Palmeae, Rubiaceae, Sterculiaceae, Solanaceae, Sapindaceae, Verbenaceae) and nineteen family with one species (Asphodeliaceae, Arecaceae, Araceae, Bromeliaceae, Combretaceae, Caricaceae, Capparaceae, Convolvulaceae, Gramineae, Leguminosae-Papilionoideae, Mimosaceae, Musaceae, Myrtaceae, Meliaceae, Rutaceae, Sapotaceae, Tiliaceae, Vitaceae). The organs most used from families are the fruits (40%), leaves (30%), bark of the trunk (9%), seeds (8%), rhizomes (5%), followed by tubers (4%) and roots (4%). The flowers (3%) are very few used.

**Table 1:** Food plants used for their fruit during wrestling time

Scientific name (Family)	Local name (Kabyè or Ewe)	Frequency	Indications
*Butyrospermum Paradoxum* (Sapotaceae)	NimKibamtuu (Kabyè)	17	Against asthma; broncho-dilating
*Cola acuminata, Cola nitada* (Sterculiaceae)	Goro (Kabyè)	13	Exciting extremely. Against migraine, neuralgia. Antidépressive
*Citrus limon, Citrus Medica* (Rutaceae)	Lamituu (kabyè)	18	Improve breathing; Anti-fatigue.
*Annona Muricata (*Annonaceae)	Tsutsurè kpolou(Kabyè), Anyigli (Ewé)	4 (6%)	Improve breathing
*Hyphaene thebaïca* (Palmeae)	Kokolay (Kabyè)	7	Antifatigue, Against Aches
*Cassia alata* (Caesalpiniaceae)	Kitchingtchig'a (Kabyè, tem)	8	Reinforce immune system. Good for nerves.
*Lycopersicon esculentum* (Solanaceae)	Timati (kabyè),	5	analgesic, give strength, Against fear
*Vitex doniana Sweet (*Verbenaceae)	Tchangbayou (Kabyè)	7	Improve breathing; Analgesic
*Arachis hypogaea Ls*. (Fabaceae)	Kètèou (Kabyè)	6	Against Aches, tirednesses
*Vigna subterranea (L.) verdc*. (Leguminosae-Papilionoideae)	Souwé (kabyè), Azîgokui (Ewé)	11	Give force, power, energy
*Cissus populnea Guill. & Perr*. (Vitaceae)	Mènyè (Kabyè)	17	Tonic
*Theobroma cacao* (Sterculaceae)	Kokoti (Ewé) Kokotuu (Kabyè)	8	Against migraine; Increase heart rate
*Cocos nucifera L*. (Arecaceae)	Anassara kpakpayè (Kabyè), Yovoneti (Ewé)	7	Tonic, improve breathing
*Citrus sinensis (L.) Osbeck*(Rutaceae)	Lémou (kabyè), N'ti (Ewé)	11	Give force, power, energy, analgesic
*Spondias mombin L*. (Anacardiaceae)	Ehilou (Kabyè), Akikoti (Ewé)	8	Antianemic, antifatigue

**Table 2:** Food plants used for their leaves and/or fruits during wrestling time

Scientific name (Family)	Local name (Kabyè or Ewe)	Frequency	Parts used	Indications
*Ananas comosus* (Bromeliaceae)	Adoto (kabyè), Agondé (Ewé)	13	Rhizome	Against infection, fever; Contains vitamins
*Allicium cepa* (Alliaceae)	Kaabou (Kabyè) Saboulè (Ewé)	18	Rhizome	Improve breathing. Against hypertension, fever. Contains vitamins
*Allium sativum* (Alliaceace)	Ayo (kabyè)	11	Rhizome	Decrease hypertension; Heal the Wound; Improve breathing; Antibiotic
*Zingiber officinale* (Zingiberaceae)	Dotè (ewé), Wissikoyè (kabyè)	21	Rhizome	Against sun negative effect. Improve breathing. Against rhumatism
*Curcuma longa L*. (Zingiberaceae)	Wissikoyè – Tchilou (kabyè),	31	Rhizome	Against rheumatism; analgesic. Improve breathing;
*Curcuma zedoaria* Roscoe (Zingiberaceae)	Wissikoyè -Kpolou (kabyè),	26	Rhizome	Stimulate nerves; improve breathing.
*Borassus aethiopum Mart*. (Arecaceae)	Kpirè (Kabyè)	27	Fruits, rhizome	Give force, power, energy
*Aloe vera*, (Asphodeliaceae)	Sinawkoyè (kabyè)	28	Leaves rhizome	Against burns, wounds, infections and pains

**Table 3:** Food plants used for their leaves, fruits and/or seeds during wrestling time

Scientific name (Family)	Local name (Kabyè or Ewe)	Frequency	Parts used	Indications
*Psidium guayava* (Myrtaceae)	Tsutsurè (Kabyè)	7	Leaves fruits	Antibiotic, antispasmodic; Improve breathing.
*Mangifera indica* (Anacardiaceae)	Mangoti (Ewé), Mangoutu (kabyè)	6	Fruit, Seeds, Leaves	Analgesic, antifatigue. Against asthma, Improve breathing.; Hallucinogen
*Caspicum frutescens* (Solanaceae)	Kpanzipié (Kabyè)	16	Fruits, Leaves	Against muscular pains, wrenches, arthritis, lumbago
*Adansonia digitata* (Bombaceae**)**	Telou (Kabyè), Adido (Ewé)	39	Fruits Leaves Seeds	Cure anaemia, fever, pulmonary affections, good for giving sensation
*Coffea sp* (Rubiaceae)	Kofe (Kabyè,Tem)	10	Seeds	Against migraine; Increase heart rate
*Musa paradisiaca* (Musaceae)	Akoroutou (Kabyè)	8	Leaves, fruits	Increase heart rate; Contains vitamins
*Parkia biglobosa* (Mimosaceae)	Soulou (Kabyè) Woti (Ewé)	14	Fruits Seeds	analgesic, give strength, against cardiac palpitation, Against fear,
*Zea mays* (Gramineae)	Bli (Ewé), Samila (kabyè)	10	Seeds	Against fever, rheumatism, cough
*Sorghum bicolor* (Poaceae)	Mirè (Kabyè)	17	Fruits, Leaves	Improve breathing
*Vigna unguiculata (L.)* (Fabaceae)	Tchassi (Kabyè)	9	Fruits, Leaves	Give force, power, energy
*Corchorus olitorius L*. (Tiliaceae)	Kuloung (Kabyè), Ademin (Ewé)	6	Leaves, fruits	Give force, power, energy, Tonic
*Blighia sapida Koenig* (Sapindaceae)	Kpiziou (Kabyè)	8	Fruits, Leaves	Analgesic, Give force, power, energy
*Hibiscus esculentus* (Malvaceae)	Maatou (Kabyè)	23	Leaves, Young fruits	antianemic

**Table 4:** Food plants used for their leaves during wrestling time

Scientific name (Family)	Local name (Kabyè or Ewe)	Frequency	Indications
*Ceiba pentandra* (L.) Gaertn. (Bombacaceae)	Comon (Kabyè)	16	Improve breathing; antianemic
*Piliostigma reticulatum (DC.) Hochst*. (Caesalpiniaceae)	Pabakou (Kabyè)	19	Against aches, tirednesses; analgesic, improve breathing
*Hibiscus sabdariffa L*. (Malvaceae)	Gnatou (kabyè)	10	Analgesic, tonic
*Acanthospermumhispidum* (Asteraceae)	Kpanzoyè (Kabyè),	17	Analgesic
*Ocimum americanum L*. (Lamiaceae)	Konzonzongo (kabyè)	9	Analgesic, antianemic
*Ocimum gratissimum L*. (Lamiaceae)	Azéou (Kabyè),	25	Analgesic, Improve breathing
*Cymbopogon citratus* (Poaceae)	Tigbé (Ewé, kabyè)	11	Analgesic; antifatigue; Stimulate perspiration. Against fever
*Vernonia Amygdalina*, (Astéraceae)	Aloma ((Ewé), Fayou (kabyè)	35	Analgesic; antifatigue; Against wounds
*Euphorbia hirta* (Euphorbiaceae)	Tchikpaltchindi (Kabyè) Rotsigbé, (Ewé)	11	Against asthma. Improve breathing. Analgésic. Anti-inflammatory drug

**Table 5:** Food plants used for their different organs during wrestling time

Scientific name (Family)	Local name ( Kabyè or Ewe)	Frequency	Parts used	Indications
*Pteleopsis suberosa* (Combretaceae)	Kizisinna (kabyè)	21	Bark	Antifatigue; against infection
*Carica papaya* (Caricaceae)	Somoré (kabyè), adibati (Ewé)	16	Leaves, fruits, Root, Seeds, Flowers	Contains vitamins. Reinforce immune system. Good for nerves
*Khaya senegalensis* (Meliaceae)	Hèmou (Kabyè), Mahougen (Ewé)	19	Bark	Improve breathing. Antianemic, Against colics, hyperthermia; Analgesic
*Tectona grandis* (Verbenaceae)	Tchalimtuu (Kabyè)	12	Leaves, Bark	Improve breathing
*Elaeis guineensis Jacq*. (Palmeae)	Pow (Kabyè)	10	Fruits, Bark, Root	Improve breathing
*Manihot esculenta* *Crantz* (Euphorbiaceae)	M'bomtiou ou bantsi (Kabyè),	11	Tuber, Leaves	Improve breathing
*Colocasia esculenta (L.) Schott* (Araceae)	Kpèkpèou (Kabyè), makali (Ewé)	8	Tuber	Tonic, Give force, power, energy
*Paullinia pinnata L*. (Sapindaceae)	Fatumagogo (Kabyè), agbassalika (ewé)	5	Stem with leaves	Analgesic, tonic, stimulant
*Bombax costatum Pellegr. & Vuillet* (Bombacaceae)	Houla (Kabyè)	22	Chalice of the flower	Give force, power, energy antianemic
*Anacardium occidentale* ssssssss(Anacardiaceae)	Yovotchati (Ewé), Atchantuu (kabyè),	13	Fruits Bark	Analgesic. Against hypertension
*Ipomoea batatas* (Convolvulaceae)	Awizèè (Kabyè),	13	Tuber	Tonic; improve breathing
*Crateva adansonï DC* (Capparaceae)	N'goné (kabyè)	25	Root	Give force, power, energy

## Discussion

Present investigation indicates that people of Togo is blessing with marvelous diversity of leafy vegetable plants. They consume and conserve the plant species for their diverse uses. The tabulated plantspecies showed that the wrestlers consumed not only the dietary values but medicines also. Most of the plants, which are enlisted in the Table 1, Table 2, Table 3, Table 4, Table 5 are widely used in the other parts of the world in similar or different ways. For example, Cocos nucifera L. [34] and Hibiscus sabdariffa L. [35] are widely used for cosmetic purposes. Among the different plant parts used in this study, leaves are secondary used after fruits. This result is not in accordance with those obtained by Jeyaprakash et al., [36], Mali & Bhadane [37] and other studies [38–40] who revealed that leaves are the most widely used plant parts. Kabyès land is located in the grassland savannah which favours the growth of herbs. Most societies and cultures have a sound knowledge of the biodiversity in their environments as a result of long term experimentation and innovation [41]. This may explain the use of many herbs in the traditional food and medicinal practice in this region. Fruits and leaves of plants have been reported to accumulate, antioxidants, vitamins, inulins, tannins and other alkaloids [42] which may be responsible for their medicinal properties, explaining their wide use. A large majority of the plants used as traditional food plants in the study areas or elsewhere in the region lacks nutritional evidences. Therefore, it is required that steps must be taken up to perform nutritional, phytochemical and pharmacological studies to support and validate the potential of local food plants. Many plant species have become threatened due to habitat loss as result rapid urbanization and other anthropogenic factors. It suggests that these findings be incorporated into future biodiversity management and valorization plans.

## Conclusion

This study reveals that many plants are used as food plants in the ground during Kabyè traditional wrestling. In view of these results, the local population must be educated on the rational use of these resources to not compromise their availability. Particular attention should be devoted to scientific research on food crops.

### What is known about this topic

- The flora is the only renewable resource for rural communities.
- Traditional healers know that plants can improve physical performance.
- Physical performance can be improved by natural substances

### What this study adds

- Some plants that can enhance physical performance but secretly kept by traditional healers, throughout Kabyè land are known.
- Some of these plants are food plants.
- Adansonia digitata is one of the most cited food plants.
